# Virtual Reality with 360-Video Storytelling in Cultural Heritage: Study of Presence, Engagement, and Immersion

**DOI:** 10.3390/s20205851

**Published:** 2020-10-16

**Authors:** Filip Škola, Selma Rizvić, Marco Cozza, Loris Barbieri, Fabio Bruno, Dimitrios Skarlatos, Fotis Liarokapis

**Affiliations:** 1 Faculty of Informatics, Masaryk University, 60200 Brno, Czechia; 2 Faculty of Electrical Engineering, University of Sarajevo, 71000 Sarajevo, Bosnia and Herzegovina; srizvic@etf.unsa.ba; 3 3DResearch S.r.l., 87036 Rende, Italy; marco.cozza@3dresearch.it (M.C.); fabio.bruno@unical.it (F.B.); 4 Department of Mechanical, Energy and Management Engineering (DIMEG), University of Calabria, 87036 Rende, Italy; loris.barbieri@unical.it; 5 Photogrammetric Vision Laboratory, Department of Civil Engineering and Geomatics, Cyprus University of Technology, 3036 Limassol, Cyprus; dimitrios.skarlatos@cut.ac.cy; 6 Research Centre on Interactive Media, Smart Systems and Emerging Technologies (RISE), 1011 Nicosia, Cyprus; f.liarokapis@rise.org.cy

**Keywords:** EEG, virtual reality, 360-video storytelling, cultural heritage, presence, immersion

## Abstract

This paper presents a combined subjective and objective evaluation of an application mixing interactive virtual reality (VR) experience with 360° storytelling. The hypothesis that the modern immersive archaeological VR application presenting cultural heritage from a submerged site would sustain high levels of presence, immersion, and general engagement was leveraged in the investigation of the user experience with both the subjective (questionnaires) and the objective (neurophysiological recording of the brain signals using electroencephalography (EEG)) evaluation methods. Participants rated the VR experience positively in the questionnaire scales for presence, immersion, and subjective judgement. High positive rating concerned also the psychological states linked to the experience (engagement, emotions, and the state of flow), and the experience was mostly free from difficulties linked to the accustomization to the VR technology (technology adoption to the head-mounted display and controllers, VR sickness). EEG results are in line with past studies examining brain responses to virtual experiences, while new results in the beta band suggest that EEG is a viable tool for future studies of presence and immersion in VR.

## 1. Introduction

Virtual reality (VR) is one of the ideal mediums for representing places that do no longer exist or that went through a radical transformation from the past. This can be leveraged especially in the case of the cultural heritage sites, which are in many cases inaccessible or with restricted access due to a variety of reasons; the heritage sites are often ruins, which are, in some cases, submerged into the sea. VR is a medium that allows for the representation of the lost sites using faithful 3D reconstructions and interactive narratives.

The strength of the experiences created using VR technology is that they feel especially “real”, thanks to the illusions arising in the virtual environments; the place illusion of “being there” (in the location depicted by the VR application) and the plausibility illusion [1]. By accepting the plausibility of the presented VR scenario (accepting that the events in the presented scenario are actually occurring), the sense of presence is conveyed. Presence corresponds to the experience of “being” in the virtual environment, despite the knowledge of being physically present at a different place and that the perceived presence is mediated using technology [1]. Immersion is responsible for creating the feeling of presence for participants in VR [2].

Subjective “realness” of the immersive VR experiences is leveraged in many fields, including science, education, and training [3]. Contrary to the real-life scenarios, VR-mediated experiences allow the inexpensive and environmentally-friendly substitution to traveling to distant places. Moreover, the knowledge acquisition is facilitated by interaction and the possibility to examine objects closely, which may not be possible in the real-life scenarios [4,5]. Clearly, maximizing the strength of the VR illusions that make the experience “believeable” is desired. Although developers of VR scenes typically aim for high levels of engagement, a sense of presence, and immersion in their applications, studies that examine these phenomena are required to fully understand the potential of VR experiences and their effects on users. Notably, there is a lack of studies providing data on the aspects of VR experiences from both subjective and objective points of view.

This paper describes the evaluation of an immersive VR experience combined with the 360° storytelling (related to the immersive VR, but lacking interactivity and translational movement in the scenes [3]) for purposes of presentation of cultural heritage. The evaluation was performed using a modern VR application created as a result of a coordinated research effort in the iMARECULTURE project [6]. In the virtual experience, users visit and explore a realistic 3D reconstruction of the underwater cultural heritage site of Baiae, recreated with the focus on a high degree of realism and the scientific soundness [7,8,9,10]. The evaluation focused on a combination of objective and subjective evaluation of the application, that was hypothesized to provide high levels of engagement. Seamless incorporation of the 360° storytelling into the “true” VR experience in the application used for evaluation was expected to provide high engagement mediated by the presence and immersion of the VR. In turn, this allowed for a detailed investigation into the subjective qualities of the experience and corresponding results recorded from brain signals.

Subjective evaluation was performed using a multi-dimensional VR user experience (VR UX) questionnaire [11], which surveys the participant responses using scales of presence, engagement, immersion, state of flow, emotional response, subjective judgment, experience consequence, and technology adoption. Presence, immersion, and technology adoption are directly related to the VR experience, while the state of flow denotes the positive state of a full dedication and concentration to the activity at hand [12]. Past research showed that the physical consequences connected to the usage of VR equipment are mostly composed of the simulator sickness [13]. The VR UX questionnaire was accompanied by the NASA-TLX questionnaire, a commonly utilized tool for investigation into the cognitive demands of tasks.

For the objective evaluation, we utilized neurophysiological measurements from the brain using electroencephalography (EEG). EEG allows scalp recording of the weak electrical currents corresponding to the neural communication in the cortex, typically using readings from multiple sensors simultaneously [14]. Reasons for the popularity of EEG evaluation in various fields of research are mainly its low cost and non-invasive approach. Commonly, the EEG signals (signatures of the brain electrical activity in time—neural oscillations, or “brain waves”) are analyzed in terms of frequency. Of special interest are the alpha waves (18–12 Hz), which play role in multiple sensory and cognitive processes, and which are known to be inversely related to attention [15,16] and cortical activation in general [17]. The beta-band oscillations (15–30 Hz) are considered to be markers of cognitive processing, especially in the upper part of the beta spectrum [16]. Theta EEG band (4–7 Hz) has been linked to the memory and cognitive performance [18].

Our results suggest that the evaluated application provided high levels of presence, engagement, and immersion. Participants did not have issues with the technology adoption in this scenario where VR and 360° storytelling were mixed in one experience, and the negative consequences of the experience remained low. Importantly, the experience was not rated as demanding by the participants, despite the incorporation of the EEG recording methodology in the session. The results in the EEG beta-band suggest increased cognitive processing by the end of the experience, reflecting the active engagement reported in questionnaires. We discuss the EEG results in the context of previous studies investigating EEG responses to VR experiences, as well as in the context of utilization of EEG in future studies in the VR research.

## 2. Background

### 2.1. Interactive Digital Storytelling

Interactive digital storytelling (IDS) is an extension of digital media-mediated narrative entertainment (termed digital storytelling [19]) where the user actively tailors the story. Various IDS methods try to maximize immersion, attractivity, and the general efficiency of the storytelling procedure, as the quality of the user experience is of the highest importance for the success of an IDS application. IDS applications face many challenges [20], one of which is the narrative paradox challenge [21], defined by the struggle between paying attention to the main storyline and the freedom of choice resulting from the interactive scenarios. This issue emerges in IDS consisting of interactive VR scenes linking stories to objects in these scenes. It is not uncommon that users miss finding the triggers, in turn missing important information. Approaches to solve the narrative paradoxes consist of important contributions to the methodology in IDS.

A solution to the narrative paradox challenge can be provided by using emergent narratives [22,23,24]; i.e., the stories emerge from the interaction between the users and the IDS systems. However, applications presenting predefined stories cannot make use of this solution. The hyper-storytelling concept [25] offers a solution based on a simpler approach, aiming to attract the users through the high quality of the storytelling and efficient information distribution, enabling them to virtually visit the 3D model of selected cultural heritage object and experience what they have been watching in stories. Guidelines for IDS [26] utilize this approach, and leverage the motivational factor as the solution for the narrative paradox.

Another challenge for IDS is to present the information on VR devices such as Head-Mounted Displays (HMDs). Users can choose their view inside the virtual environment, so the rules of film language grammar and shot composition do not apply anymore. There are several projects using 360° videos for communicating the cultural heritage information. The conceptual gamification framework for VR applications was proposed by Argyriou et al., (2017) [27]. They enhanced user interaction in a case study of the cultural heritage site in Rethymno city (Greece) by mixing game elements into 360° videos storytelling. After presenting the 360° videos to convey information, the user was presented with a quiz and a motivational factor. This approach benefits from high replayability value, but in case the game was played only once, only a subset of all the information would be presented to the user.

### 2.2. Underwater Virtual Reality

It has already been demonstrated that the usage of VR is effective for increasing of the cultural heritage impact [28,29,30,31,32,33]. The same applies for AR where the motivation for cultural heritage has been recently documented [34]. However, the actual applications in the underwater cultural heritage remained mostly unexplored, until recently. In an early approach, issues were discussed regarding the virtual access of underwater archaeology for the non-diving public [35]. Moreover, frameworks for the collection and visualization of the underwater assets have been proposed, but only a single archaeological site could be exploited with this approach [36]. Alternatively, the existing applications have been focused on the scientific purposes (rather than edutainment of the general public) [37]. AR has also been exploited for improving of the diving experience in Underwater Archaeological Park of Baiae, in a study performed in scope of the iMARECULTURE project [38].

The Amphibian system from MIT provided an immersive VR SCUBA diving experience by simulating buoyancy, drag and temperature changes through a variety of sensors [39]. In terms of VR reconstruction of the underwater archaeological sites The Virtual Exploration of Underwater Site (VENUS) project [40] pioneered. Several VR and AR tools have been focused to aid the archaeologist study of the virtual sites using interactive and immersive visualization [41]. Re-adapted versions of the VR-based demonstrations have been created, but without the goal of edutainment, focused rather on the presentation and visualization [42]. One of the few exceptions adopting an edutainment approach to educate about the underwater cultural heritage applied a serious game design to teach about the protection of global oceanic resources with the focus on an elementary school audience [43].

Liarokapis et al., (2017) [44] proposed an underwater VR reconstruction and visualisation of the Mazotos shipwreck site to raise the archaeological knowledge. The application makes use of procedurally generated artefacts, flora, and the rest of the underwater environment. The VISAS project [9] is a diving simulation in VR allowing users to experience a simulation of a real diving session from the perspective of the scuba diver. In the application, a virtual companion guides the user through an exploration of the submerged archaeological sites, developed with a high degree of realism. This is complemented with a general and historical–cultural context as well as with information regarding the flora and fauna of the underwater site.

Recently, an archaeological VR application simulating diving into the reconstructions of several submerged sites combined with interactive storytelling, has been proposed in the scope of the iMARECULTURE project [6,26,45]. The 360° videos are used to convey the digital storytelling, and the option to switch between the realistic underwater reconstruction and a hypothetical depiction of the original state of the site helps to enhance the strength of the experience.

Immersive VR for teaching the future marine archaeologists about the essentials of the underwater excavation (i.e., operating an airlift) was developed recently [46]. The environment includes simulations of fog and caustics to faithfully mimic the underwater lighting conditions. An educational VR application aiming to aid the future marine archaeologists with the basics of photogrammetry was developed by [47]. This gamification technique has proven useful in the creation of accurate measurements, according to the initial results. Finally, Beacon Virtua presented the recent and shipwreck history of Beacon Island [48] using VR technologies allowing visitors to virtually experience the site. However, no formal user testing was performed, and only informal feedback was collected.

## 3. Archaeological VR Application

### 3.1. System Architecture

The architecture of the evaluated archaeological VR application is built from the four following parts: a Scene Editor module, a Database (DB), a Web Service, and a VR interaction and visualization module. Scene Editor (as well as the VR modules) were implemented using the cross-platform game engine Unity. Usage of the Unity framework is beneficial due to its simple programming of the web-based applications (using the web service software) with the data manipulation implemented using a dedicated database. The Scene Editor module was used to create the virtual scenario by integrating the data saved in the DB (3D models, multi-medial data), while the communication between the DB and the rest of the modules was implemented using the Web Service module. The key elements composing the virtual scenes were the 3D models reconstructing the “Villa con ingresso a protiro” complemented by the POI elements (represented with 3D models of large map pins).

The VR module of the archaeological application implements the logic of the virtual scenario, defines the physics and other behaviors in the VR environment. Additionally, the mechanisms allowing the exploration of the environment are implemented there. To make the VR scene visually and behaviorally realistic, accurate simulations of physics (light rays, refractions, fog, caustics, particles, and bubbles) have been implemented. The VR environment also contains models of the fauna and flora typical for the specific underwater ecosystem. That concerns specifically models of the fish and schools of fish, animated using artificial intelligence techniques, as well as realistic representation and behavior of the underwater vegetation. For a detailed description of the development and methods used in the evaluated archaeological VR application, please see the paper [10].

### 3.2. Apparatus

The application is presented in a VR system utilizing the HMD. This technology has been selected for its ability to provide high levels of immersion [3], by separating the user from potential distractions created in the physical environment and presenting the virtual scenes in a wide field of view, incorporating the peripheral space. The utilized HMD is HTC Vive headset with a resolution of 1080 × 1200 per eye, 90 Hz refresh rate, and a field of view of 110°. HTC Vive is coupled with wireless handheld motion-tracked controllers and a laser positioning technology that provides six degrees of freedom tracking up to 4.5 m × 4.5 m area with two base stations.

In the HMD, the user experiences the immersive VR environment from the first-person view of the scuba diver simulating a real diving session that starts above the water surface in the diving spot. To enhance the immersion, virtual versions of the HTC Vive controllers (with the same appearance, position, and orientation) are replicated in the virtual scene. The orientation of the controller is leveraged to move the user in the desired direction in the process of exploration of the underwater part of the application, while the visual point-of-view is controlled by the position and orientation of the HMD. Realistic reconstruction of the terrestrial environment is also present in the scene, created with the focus on a high degree of realism. Moreover, the VR scene contains a buoy, an inflatable boat, and the stretch of the coastline overlooking the diving site.

### 3.3. User Interaction with the Application

**Figure 1:**
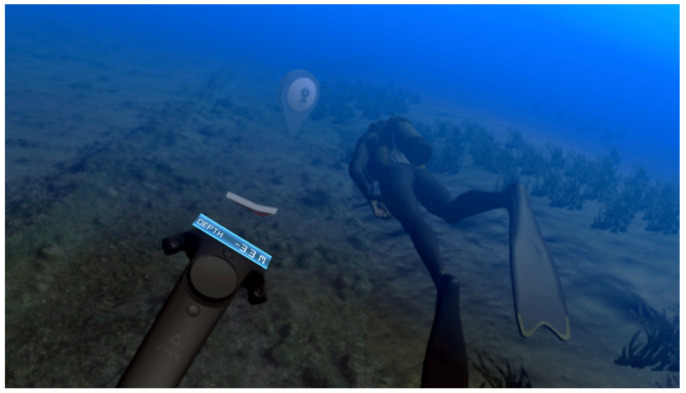
User while following the diver guide in the underwater part of the archaeological VR application.

The user activates the “swimming” by pulling the trigger of the Vive controller, and the direction of the movement is determined by the pointing direction of the controller. If the trigger is pressed all the way down, the speed of swimming is increased. After submerging into the underwater environment (Figure 1), a 3D arrow indicates the desired direction in order to reach the virtual scuba diving companion guiding the user and a label informs the user about the depth from the surface of the water.

**Figure 2:**
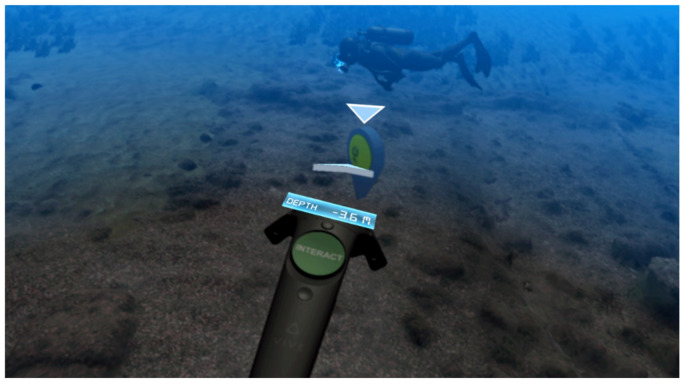
The POI becomes active when the user approaches it.

As depicted in Figure 2, when the user is in the immediate proximity of a POI, the controller’s trackpad becomes active and a message informs the user that it is possible to activate the POI. If activated, the related 360° video begins playing automatically in the HMD. Apart from the first video, all of them are set in the hypothetical reconstruction of the original state of the “Villa con ingresso a protiro”, while they feature real actors of appropriate ages for playing the characters and resembling Romans [26]. When the user is in this video mode he/she can explore the scene by turning his/her head, and the user can press the trigger on the Vive controller to return to the 3D underwater virtual scenario.

When the user is guided to the final POI by interacting with it, he/she can switch from the wet to a dry environment that consists of a suggestive 3D reconstruction of the architectures not existing anymore. The user can then “walk” into the Villa and explore its original magnificence. In the dry 3D environment, the user interaction occurs in the same way as described for the underwater scenario, the only difference is that the user cannot move as in the water. The 3D hypothetical reconstruction has been carried out through a theoretical and multidisciplinary scientific approach [49] on the basis of archaeological evidence and, for the parts that did not survive to the present, proposed reconstructions are based on the coeval examples known from the literature.

As mentioned above, when the guided tour is finished, the user has the possibility to freely explore the archaeological area and to select the desired POI and interact with it. Differently from the POI that enables 360° videos, the underwater scene contains also POIs providing a textual and audio description. The interaction has a similar form, but the visual content is displayed in a 3D frame within the VR scene.

### 3.4. VR Storytelling

The storytelling for Baiae archaeological VR application was created according to the Sarajevo Charter guidelines [26]. We encountered a particular challenge to produce the stories in HMD-mediated VR video when the user is free to look around while watching the story, so the rules of shot composition and staging from film language grammar do not apply anymore.

The interactive visit of the archaeological site is enriched by the story of its hypothetical owner, Gaius Vibius Sabinus, an aristocrat from an important Roman family. He is the owner of the villa and a part of the Baiae coastline on the Lacus Baianus, where he spends his time with his family and friends, relaxing. He is a passionate collector of Greek art as well as well-done copies. Local artist and copyist Heliodorus and his apprentice Saturninus visit the aristocrat in his villa and discuss his intention to purchase a statue.

**Figure 3:**
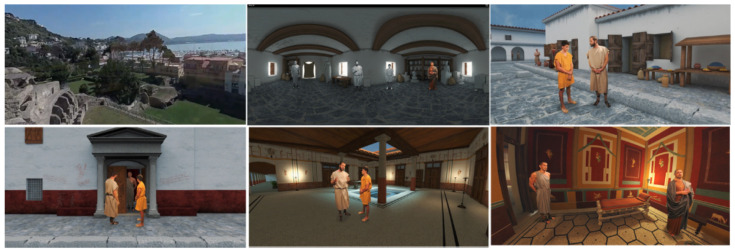
Screenshots of the 360° storytelling scenes in the archaeological VR application.

**Figure 4:**
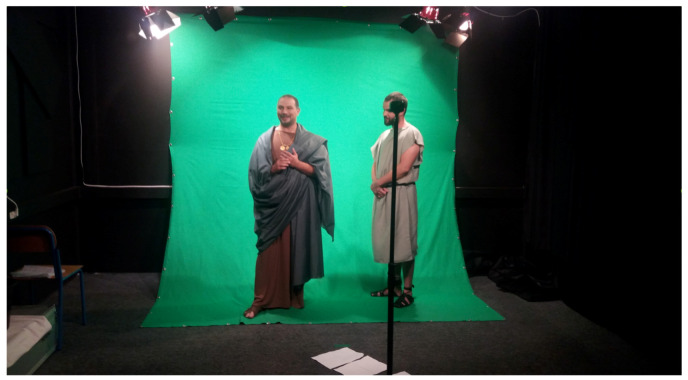
Recording of actors for the 360° VR storytelling (a written consent for the publication of the photos has been obtained from the actors).

The storytelling was conceived and written by experts, and it consists of six parts: the introductory story (360° video of Baiae remains on the land with a voice explaining to the user the historical significance of the city), Heliodorus’ workshop (the characters are introduced there; Heliodorus—the sculptor, Saturninus—his apprentice, Gaius Vibius Sabinus—the aristocrat, and Serapis—the slave), the street with shops, the entrance to the villa where Heliodorus and Saturninus are introduced, the room with mosaics, the atrium, and the final scene including the discussion of Heliodorus and Sabinus about the design and price of the statue. See Figure 3 for screenshots taken from the application. Stories in the intro story were created using 360° camera recordings, and in the rest of the application, 360° renders from 3D scenes were composited with actors recordings against the green screen background [50] © 2019 IEEE (Figure 4).

## 4. Materials and Methods

### 4.1. Participants

From the original 16 volunteers participating in the study, one had to be excluded due to a technical failure in the EEG recording, resulting in the number of participants N = 15 (10 male, 5 female). The mean age of the participants was 26.6 (SD = 2.293). All participants had some experience with HMD-mediated VR (median reported VR experience 3 on scale 1–5; SD = 0.799). The study was approved by the Research Ethics Committee of the Masaryk University, and all the participants gave their written consent to participate in the study.

### 4.2. Procedure

After being informed about the experiment, giving their consent to participate, and filling-in the pre-experimental questionnaires, the usage of the VR system was explained to the participants. Specifically, the inter–ocular distance of the HMD was customized, and the usage of controllers was explained. Then the participant was seated on a swivel chair allowing customization of the height, where the EEG device was set-up (taking approximately 15 min). EEG set-up consisted of placing the electrode cap on the head, application of the conductive gel to each of the electrodes, and checking the impedance. After the EEG signals were of satisfactory quality (the noise levels were low), the HMD was placed on the participant with the help of the experimenter, and the signals were re-checked to make sure the HMD did not interfere with some of the electrodes. As the last step, the participant received headphones and the VR controllers.

Participants interacted with all the POIs in the VR application. The experiment was finished after the last POI was attended (participants did not interact with the dry reconstruction of the Villa after the last POI was attended). In total, the VR experience took approximately 12 min. At the end of the experimental session, the equipment was taken off (headphones, HMD, EEG), and participants were asked to fill-in the VR UX and NASA-TLX questionnaires. The total duration of the experiment was approximately 60 min.

### 4.3. EEG Data Collection

EEG was collected using a lightweight EEG system Neuroelectrics Enobio 32 using 8 gel-based AgCl electrodes referenced to the right earlobe. Electrode montage concerned the pre/frontal (FPz, F3, F4, Fz), parietal (P3, P4, Pz), and occipital (Oz) regions (the electrode names follow the international 10/20 system for EEG recording [51]).

### 4.4. Questionnaires

For the evaluation of the subjective responses to the VR experience, two questionnaires were used; VR UX questionnaire [11] focused on the user experience, and the NASA Task Load Index (TLX) [52] focused on cognitive demands. The VR UX questionnaire is the result of a compilation of several well-known questionnaires, and consequently surveys the user experience in eight sub-scales; presence, engagement, immersion, flow, emotion, judgment, experience consequence (VR-related sickness), and the technology adoption (focused on the controllers usage). Answers were positioned on a 7-point Likert scale ranging from −3 (“Not at all”), through 0 (“Somewhat”), to +3 (“Completely”). The NASA-TLX is answered on a 21-point scale. Apart from these two questionnaires, we also surveyed participants’ demographics, their experience with VR (one-item self-evaluation), and the time spent with computers daily.

### 4.5. Data Analysis

The recorded data were analyzed using EEGLAB [53]. In the preprocessing phase, the signals were downsampled to 100 Hz to clean the 50 Hz power line noise, and high-pass filtered with cut-off frequency 1.5 Hz. To clean the data, we applied artifact subspace reconstruction algorithm [54]. The occipital region (EEG channel Oz) was discarded from the analysis due to a large number of artifacts at this electrode site. To prevent biasing the results towards stronger power in the frontal region, the channel FPz was excluded for the reasons of absolute differences in the spectral power between this channel and the rest of the region in most participants. Cleaned data were re-referenced to full-rank average reference and processed with the independent component analysis (AMICA implementation) to differentiate artifactual sources in the signal from the EEG signals originating from the brain. Removal of the artifactual components was automatized using a multiple-artifact rejection algorithm [55] (available as a plugin for EEGLAB).

For the subsequent analysis, three epochs of the EEG data were produced; the baseline (preceding the VR experience, with HMD worn), the early phase (created from the very beginning of the VR experience, the initial storytelling phase), and the final phase (created from the last phases of the storytelling). Only storytelling phases in the VR application were used for the EEG analysis purposes due to none or low bodily movement produced while observing the virtual storytelling, while all the epochs were 15 s long.

Power spectral density from each of the three epochs were generated using spectopo function in EEGLAB. These values were subsequently delogarithmized to produce absolute band powers per the three EEG bands of interest; theta oscillations (4–8 Hz), alpha oscillations (8–12 Hz), and the higher part of the beta spectrum (16–30 Hz). For purposes of finding out the effect of the VR exposure to the EEG spectral indices, within-subject differences in absolute band powers (in baseline, initial phase, and the last phase) were tested. To compute correlations between the EEG results and the questionnaires, the index of neural de/synchronization was computed as a percentage change in the absolute band power between the baseline and the final phase.

## 5. Results

### 5.1. Questionnaire Results

Descriptive statistics from each of the VR UX questionnaire sub-scales indicated high adaptation to the virtual experience. All the positive sub-scales (presence, engagement, immersion, flow, emotion, judgement, and technology adoption) had their medians greater or equal to +1 rating in the 7-point Likert scale (this is true also for the average responses with the exception of judgment with mean answer equal to +0.978). The experience consequence sub-scale was rated with the median answer −2.5.

**Figure 5:**
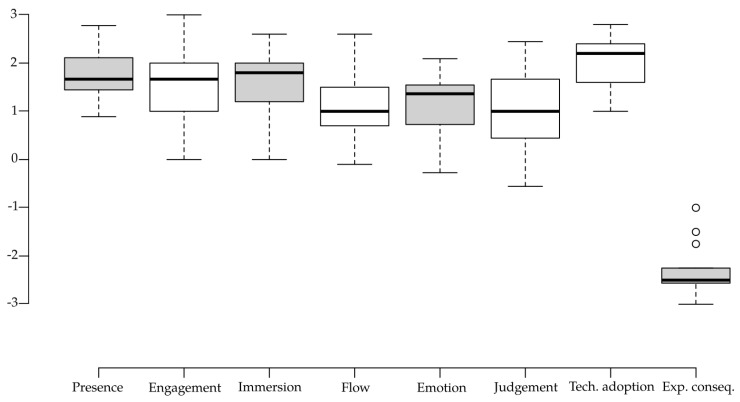
Boxplot showing descriptive statistics for each of the VR UX subscales.

**Figure 6:**
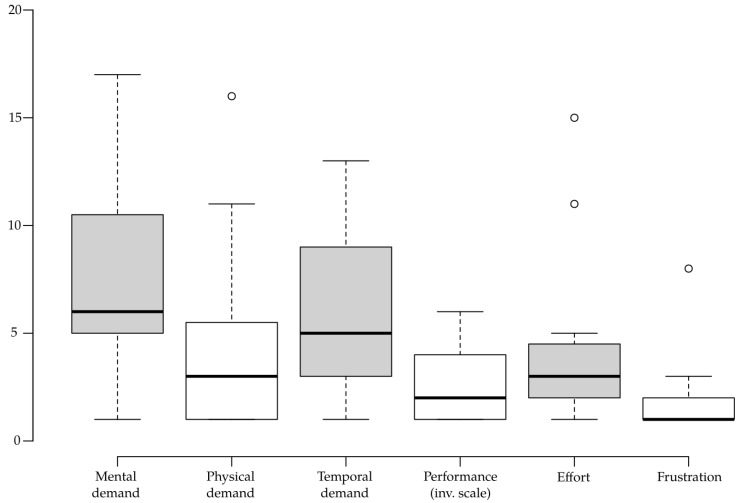
Boxplot showing descriptive statistics for each of the NASA-TLX subscales.

Most importantly, the results show high levels of presence (mean = 1.740, SD = 0.558), engagement (mean = 1.511, SD = 0.825), and immersion (mean = 1.560, SD = 0.764). Results of all the VR UX sub-scales are visualized in Figure 5, and the descriptives for raw NASA-TLX results are shown in Figure 6.

### 5.2. EEG Results

Previous VR studies using EEG evaluation demonstrated increased alpha and theta band power following the VR exposure (see Section 6 for more details). These findings were confirmed. Moreover, exposure to the VR application was accompanied by an increase in the high beta band powers.

#### 5.2.1. Frontal EEG

Band power in the frontal theta EEG band was significantly higher during the virtual experience (comparison to the baseline), in both the initial (Z = −3.181, *p* = 0.001) and the final (Z = −1.988, *p* = 0.047) phase (see the details on the descriptive statistics in Table 1). Alpha power increase reached statistical significance in the initial phase (Z = −2.613, *p* = 0.009), while the significance was borderline in the final phase (Z = −1.704, *p* = 0.088).

Finally, the high beta band power was significantly increased in the latter phase of the EEG measurement (Z = −2.215, *p* = 0.027), while the difference between the baseline and the initial phase was inconclusive (Z = −0.909, *p* = 0.363).

#### 5.2.2. Parietal EEG

The baseline parietal theta significantly differed to the final phase (Z = −2.783, *p* = 0.005), as well as to the initial phase (Z = −2.385, *p* = 0.017). The final phase alpha power differed from the baseline with a borderline significance (Z = −1.817, *p* = 0.069). No differences were found in the parietal beta.

### 5.3. Correlations

Pairs of the sub-scales in VR UX questionnaire were correlated in the number of cases; for clarity, these correlations are presented in Table 2. Very strong correlations between VR UX and NASA TLX questionnaires were found between the experience consequences and both the temporal demand (r = 0.726, *p* = 0.002) and the physical demand (r = 0.650, *p* = 0.009). Moreover, the total duration of the exposure to the archaeological VR application negatively correlated to the technology adoption (r = −0.576, *p* = 0.025), negatively to immersion (r = −0.547, *p* = 0.035), and positively to the mental demand (r = 0.696, *p* = 0.004), showing that participants who needed more time to explore the VR reported lower quality of the experience. This indicates that longer stays in the VR were due to poor familiarization with the technology and low engagement, rather than due to participants enjoying the experience and choosing to spend more time inside the virtual environment.

## 6. Discussion

We performed an EEG and questionnaire evaluation of a novel VR application seamlessly combining an interactive archaeological VR visit to a submerged underwater archaeological site with the immersive 360° video storytelling. Results confirm a high positive rating of the subjective scales linked to the VR experience, such as presence, engagement, and immersion. EEG spectral results show a trend towards the increased band power in all the examined bands (theta, alpha, upper beta), with the significance of the beta band, showing increased cognitive processing.

Subjectively, participants rated the tested application with high scores in multiple dimensions of the VR UX questionnaire, indicating their positive adaptation and engagement in the combined VR and storytelling experience. None of the participants was naïve to HMD-mediated VR (majority [60%] of the participants were somewhat familiar to VR with rating 3 out of 5), which rules out a strong influence of the novelty and “wow” effects from the immersive technology. NASA-TLX revealed that some participants considered the experiment mentally and temporally demanding, but it is important to bear in mind that extra demands were created by the accompanied EEG testing. Indeed, the EEG evaluation, despite its non-invasive and painless nature, increases the demands on the participants in terms of time (the experiment is usually longer than the actual testing phase) and comfort (conductive gel is applied to the scalp). Still, the predominantly positive responses in the VR UX questionnaire show that despite some increases in the perceived demands, this did not prevent participants from having an immersive and engaging VR experience.

Absolute band power in all the studied EEG bands increased in the span of the VR experience, which is not a common result of experimental intervention to the EEG spectral properties. In general, an increase in the alpha band has been linked to inactivity of the underlying brain areas and it is used as a marker of inattention [16], while an increase in beta is linked to cognitive processing, and an increase in theta is related to memory processing [56]. Thus, a direct interpretation of the EEG results (participants were in a state of an increased cognitive and memory processing, but inattentive at the same time) would be somewhat contradicting. Nonetheless, similar results are common in EEG VR studies. Previous research demonstrated increases in the absolute band power (mainly theta and alpha in [57]) in response to a VR exposure. Clemente et al., (2014) [58] found an increase in alpha and theta band powers while navigating in a virtual environment (VR was mediated by a powerwall screen), and Kim et al., (2019) [57] confirmed an increase in the alpha and theta between baseline and post-exposure resting-state EEG after watching 360° videos. Frontal theta was increased during the encoding phase of a VR route presentation in [59].

Although our EEG set-up did not allow for explanation of the alpha and theta band power changes, they were used for validation of the utilized EEG methodology. This study confirms the past findings and extends them to (a) VR set-up with high immersion (using HMD); and (b) more strict EEG recording methodology (both the baseline and the active phases of EEG data were recorded with the HMD being worn, and both of the active phases were recorded during the VR exposure, not after its completion).

While alpha and theta band power increases served for validation of our EEG recording methodology, the previously unreported increases in beta band power were ascribed to the actual experimental intervention; i.e., participants were in a state of heightened cognitive processing as a result of the VR experience. Specifically, we interpret high beta band powers as markers of the engaging experience which employed users’ cognitive resources.

### Limitations

This study employed a relatively low number of participants (N = 15). Nevertheless, due to the logistical restrictions imposed by the evaluation with an EEG device, the number of participants should be considered satisfactory. Due to the poor spatial resolution and signal-to-noise ratio of EEG, the brain origins of EEG signals are difficult to disentangle from contaminators of the signal. Our EEG set-up allowed the evaluation of the band powers during the VR experience, but an EEG set-up with a higher number of electrodes and more strictly controlled environment will be needed to establish neurophysiological correlates, especially with respect to the cortical sources of the EEG activity [60].

## 7. Conclusions

An interactive underwater archaeological VR application combined with the 360° storytelling was evaluated with 15 participants, using both questionnaires and physiological readings using EEG. Results provide evidence that the application was well-accepted by the participants, sustaining high levels of immersion and engagement into the experience, while showing a state of increased cognitive processing in the EEG readings. More specifically, participants rated the application positively in scales concerning the strength and “believability” of the VR experience (presence, immersion, subjective judgement), as well as its technological implementation and lack of negative consequences (technology adoption, VR experience consequence). The EEG readings are in line with past literature, while the novel results in the beta band (an increase of band power) were identified as markers of the subjectively engaging VR experience.

Results gathered from the VR UX and NASA-TLX questionnaires also demonstrate that high levels of immersion, engagement, and other properties relevant to a quality VR experience can be maintained even when the EEG measurement is part of the methodology. This suggests that EEG is a feasible objective method for evaluation of the VR experiences, with future prospects for replacing the lengthy and inaccurate subjective evaluation methods.

## Figures and Tables

**Table 1:** Band power of analyzed EEG band; units are in dB, SD is in parenthesis.

EEG Band	Baseline	Initial	Final
Frontal Theta	1.112 (0.845)	1.887 (1.383)	1.383 (0.594)
Frontal Alpha	0.502 (0.278)	0.602 (0.323)	0.597 (0.261)
Frontal High beta	0.197 (0.096)	0.232 (0.132)	0.247 (0.123)
Parietal Theta	0.917 (0.416)	1.273 (0.465)	1.526 (0.748)
Parietal Alpha	0.580 (0.480)	0.695 (0.275)	0.705 (0.273)
Parietal High beta	0.293 (0.175)	0.301 (0.172)	0.303 (0.145)

**Table 2:** Correlation coefficients and *p*-values (in parenthesis) between the pairs of questions in VR UX questionnaire (only correlations with *p* < 0.05 are shown).

Scale	Presence	Engagement	Immersion	Flow
Engagement	0.796 (0.000)	-	-	-
Immersion	0.540 (0.038)	0.651 (0.009)	-	-
Flow	-	-	0.767 (0.001)	-
Emotion	0.562 (0.029)	0.613 (0.015)	0.621 (0.013)	-
Techn. adoption	-	0.589 (0.021)	0.780 (0.001)	0.606 (0.017)
Judgement	0.596 (0.019)	0.760 (0.001)	0.800 (0.000)	0.567 (0.027)
Judgement	and emotion	0.653 (0.008)
	and tech. ad.	0.576 (0.025)		
